# Morphological and molecular characterization of *Butlerius butleri* Goodey, 1929 (Nematoda: Diplogastridae) from South Africa: First report

**DOI:** 10.21307/jofnem-2021-026

**Published:** 2021-03-26

**Authors:** Chantelle Girgan, Gerhard Du Preez, Hendrika Fourie, Milad Rashidifard

**Affiliations:** 1 Nematology Unit, Biosystematics, ARC-Plant Health and Protection, Private Bag X134, Queenswood, 0121, South Africa; 2 Unit for Environmental Sciences and Management, North-West University, Private Bag X6001, Potchefstroom, 2520, South Africa

**Keywords:** *Butlerius butleri*, Compost, First report, Morphology, Morphometrics, Molecular biology, Systematics, Taxonomy

## Abstract

Two populations of a *Butlerius* species were recovered from compost in two gardens in Potchefstroom, North-West Province, South Africa. Although the genus has previously been reported from South Africa, no species of the genus has ever been identified in the country. Based on morphological, morphometric, and molecular studies, the specimens were identified as *Butlerius butleri* and are herein reported for the first time from South Africa. The South African specimens are 1,082 to 1,423 µm long, *a* = 40.8 to 47.6; *b* = 4.7 to 5.8; *c* = 4.0 to 6.0; *c′* = 117 to 16.3; *V* = 44 to 47%. Cuticle with evenly spaced punctations. Reproductive system didelphic, amphidelphic, both branches equal in length. Four large glands opening into proximal part of uterus. Males with prominent sphincter present in mid-region of *vas deferens*. Spicules 36 to 43 μm long, gubernaculum 23 to 31 µm long, nine pairs of genital papillae, three pre-cloacal and six post-cloacal, formula: v1, v2, v3d/v4, ad, ph, v5, 6, 7, pd. The v5, 6, 7 clusters greatly separated, left subventral group at level of phasmid, right subventral group at level of posterior dorsal papilla. Although there were some differences, the South African populations of the species compare well to all know descriptions of the species. Phylogenetic analysis based on partial small subunit (SSU) rDNA sequences showed that both South African populations of *B. butleri* are in a maximally supported sister relation with an Iranian population of this species. Based on large subunit (LSU) rDNA sequences, the two populations of *B. butleri* clustered together in a well-supported clade.

*Butlerius* Goodey, 1929 is a nematode predator belonging to the family Diplogastridae (Micoletzky, 1922). According to Sudhaus and Fürst von Lieven (2003), there are 15 valid species within the genus. The same species were also listed as valid by Bezerra et al. (2021) on the Nemys platform. However, Andrássy (2005) listed only 10 of these species as valid and according to Shokoohi et al. (2015) only 13 species within the genus are valid. The latter followed in this paper. *Butlerius* has been reported from Africa, Asia, Europe, North America, and South America inhabiting soil, compost, moss, and rotting plant material (Andrássy, 2005; Heyns, 1971). *Butlerius butleri* Goodey, 1929 type species of the genus and was described from rotted banana roots based on only five females, two males, and two juveniles (Goodey, 1929). The species was redescribed by Ahmad et al. (2009) from specimens found in rotting plant material in South Korea. More recently Shokoohi et al. (2015) described a population from vermicompost in Iran and presented the first molecular characterization of the species.

In South Africa the genus *Butlerius* was first reported in 1961 (Heyns, 1961) and are commonly found in soil samples (Dr Marais, ARC-PHP, personal communication). However, populations of the genus are very rarely formally reported in the country. Species of the genus are yet to be reported or described from South Africa. During sampling of compost matter from two different localities in Potchefstroom, North-West Province, South Africa, two populations of the genus *Butlerius* were found and identified as *B. butleri* and is herein reported for the first time from South Africa using morphological and molecular techniques.

## Materials and methods

### Sampling, nematode extraction, and fixation

Soil samples were collected from two home garden compost heaps (garden 1/population 1: 26°42′25.3″S 27°06′25.9″E; garden 2/population 2: 26°41′17.4″S 27°06′05.6″E) in Potchefstroom, North-West Province South Africa. Following, the samples were transported in cooler boxes to the North-West University and stored at 6 to 8°C until further processing. Nematodes were extracted from soil samples using the adapted decanting and sieving followed by the sugar centrifugal-flotation method (Marais et al., 2017). The extracted nematodes were fixed in a heated 4% formaldehyde plus 1% propionic acid (FPG) solution, dehydrated in a glycerin solution following De Grisse (1969) and mounted in glycerin on glass microscope slides.

### Morphological characterization

Measurements and drawings of the mounted specimens were done with a Zeiss Axio Imager A2 equipped with an Axiocam ERc5s camera and iPad with the Labscope imaging application as a drawing tube. All measurements and identifications were done at 1,000 × magnification. Curved structures were measured along the median line. All measurements in the descriptions are given in micrometers (µm) and in the form: mean ± standard deviation (range). All specimens were deposited in the National Collection of Nematodes (NCN), Biosystematics, Agricultural Research Council – Plant Health and Protection (ARC-PHP), Pretoria.

### DNA extraction and PCR

One specimen from each population was transferred into an Eppendorf tube containing 15 µl of ddH_2_O and DNA was extracted from each population using the chelex method (Musapa et al. 2013) modified by Rashidifard et al. (2019) as follow: 20 μl chelex-100 (5% w/v) and 5 μl proteinase K (20 mg ml^−1^) were added to each tube containing the nematodes, the tubes were vortexed (15 sec) and centrifuged at 8,000 rpm for 10 sec. Finally, the tubes were incubated at 56°C for 2 hr followed by incubation at 95°C for 10 min before they were stored at −20°C. The following DNA markers were used to amplify the small and large sub units (SSU and LSU) genes, respectively: SSU F04 (5′-GCTTGTCTCAAAGATTAAGCC-3′), SSU R26 (5′-CATTCTTGGCAAATGCTTTCG-3′) (Blaxter et al., 1998), and D2A (5′-ACAAGTACCGTGAGGGAAAGTTG-3′), D3B (5′-TCGGAAGGAACCAGCTACTA-3′) (Subbotin et al., 2006). Following DNA extraction, the polymerase chain reaction (PCR) was carried out using an Eppendorf Mastercycler gradient thermal cycler (Eppendorf, Hamburg, Germany); more details are provided in Table 1. The amplification tube contained 12.5 μl master mix (Promega Corporation, USA), 1 μl of each of the primers (i.e. forward and reverse), 5 μl DNA, and 5.5 μl ddH_2_O.

**Table 1.** Polymerase chain reaction steps used for amplification of the SSU and LSU rDNA genes.

		35 cycles			
Primers	Initial denaturation	Denaturation	Annealing	Extension	Final extension
SSU F04/SSU R26	94°C 3 min	94°C 45 sec	54°C 45 sec	72°C 45 sec	72°C 6 min
D2A/D3B	94°C 3 min	94°C 45 sec	56°C 45 sec	72°C 45 sec	72°C 6 min

Four microliters of PCR product were loaded on a 1% agarose gel to check the DNA quality. The DNA was stained using GelRed and then visualized under a UV transilluminator. The PCR product was stored at −20°C before sequencing by inqaba biotec™, South Africa (www.inqaba-southafrica.co.za).

### Phylogenetic analyses

Selection of the appropriate taxa for SSU phylogenetic analysis was done according to Shokoohi et al. (2015). For the LSU analysis the available sequences for Diplogastridae, as well as the outgroups, were obtained from NCBI GenBank. The sequences of selected taxa for each gene were aligned using the MUSCLE tool (Edgar, 2004) implemented in Geneious Prime® 2019.2.1 (https://www.geneious.com). Post-editing for each alignment was done using Gblock program (version 0.91b) (http://phylogeny.lirmm.fr/phylo_cgi/one_task.cgi?task_type=gblocks) with all three less stringent parameters. The jModelTest program 2.1.10 (Darriba et al., 2012) was used to identify the best nucleotide substitution model. General time reversible model with a Gamma distribution (GTR + G) was selected as the most appropriate model for SSU and D2-D3 LSU analyses. Bayesian inference (BI) was performed using MrBayes v3.1.2 (Ronquist and Huelsenbeck, 2003) Geneious Prime® 2019.2.1 and the chains were running for 2 × 10^6^ generations for both the SSU and LSU datasets. After discarding a 25% burn-in sample to estimate the posterior probabilities of the phylogenetic trees, the Markov Chain Monte Carlo (MCMC) algorithm was used (Larget and Simon, 1999) to estimate the posterior probabilities (PI) with the 50% majority rule. One population of *Odontopharynx longicaudata* De Man, 1912 was selected as outgroup for each dataset.

## Results

### *Butlerius butleri* (Goodey, 1929)

Figures 1-3

**Figure 1:**
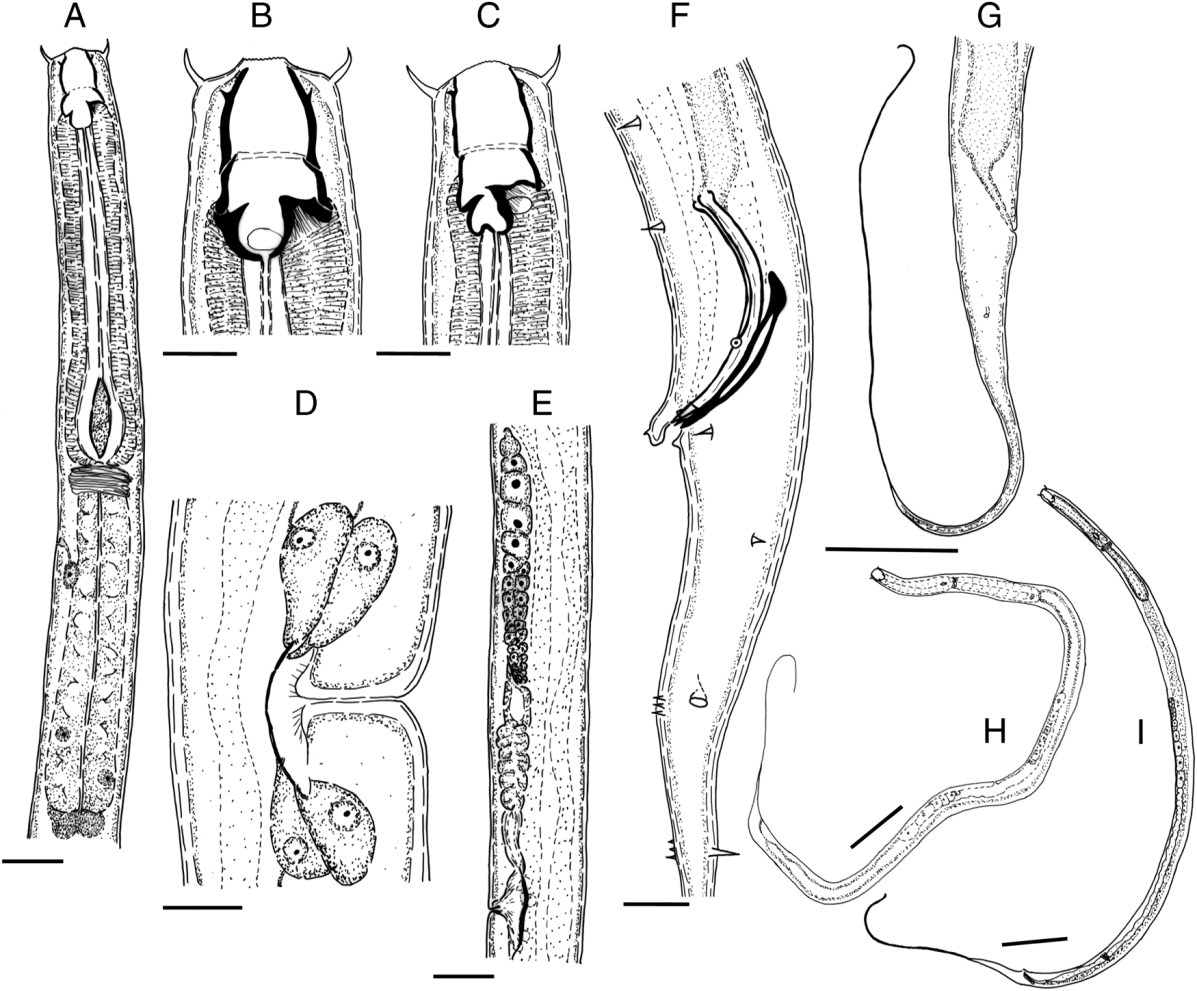
*Butlerius butleri*. A: Female anterior region; B: Female stoma; C: Male stoma; D: Vulval region indicating four large glands opening into proximal part of uterus; E: Anterior branch of female genital tract; F: Male posterior region indicating genital papillae; G: Female tail; H: Female habitus; I: Male habitus. Scale bars: B to D, F, H: 10 µm; A, E: 20 µm; G: 50 µm; H, I: 100 µm.

**Figure 2:**
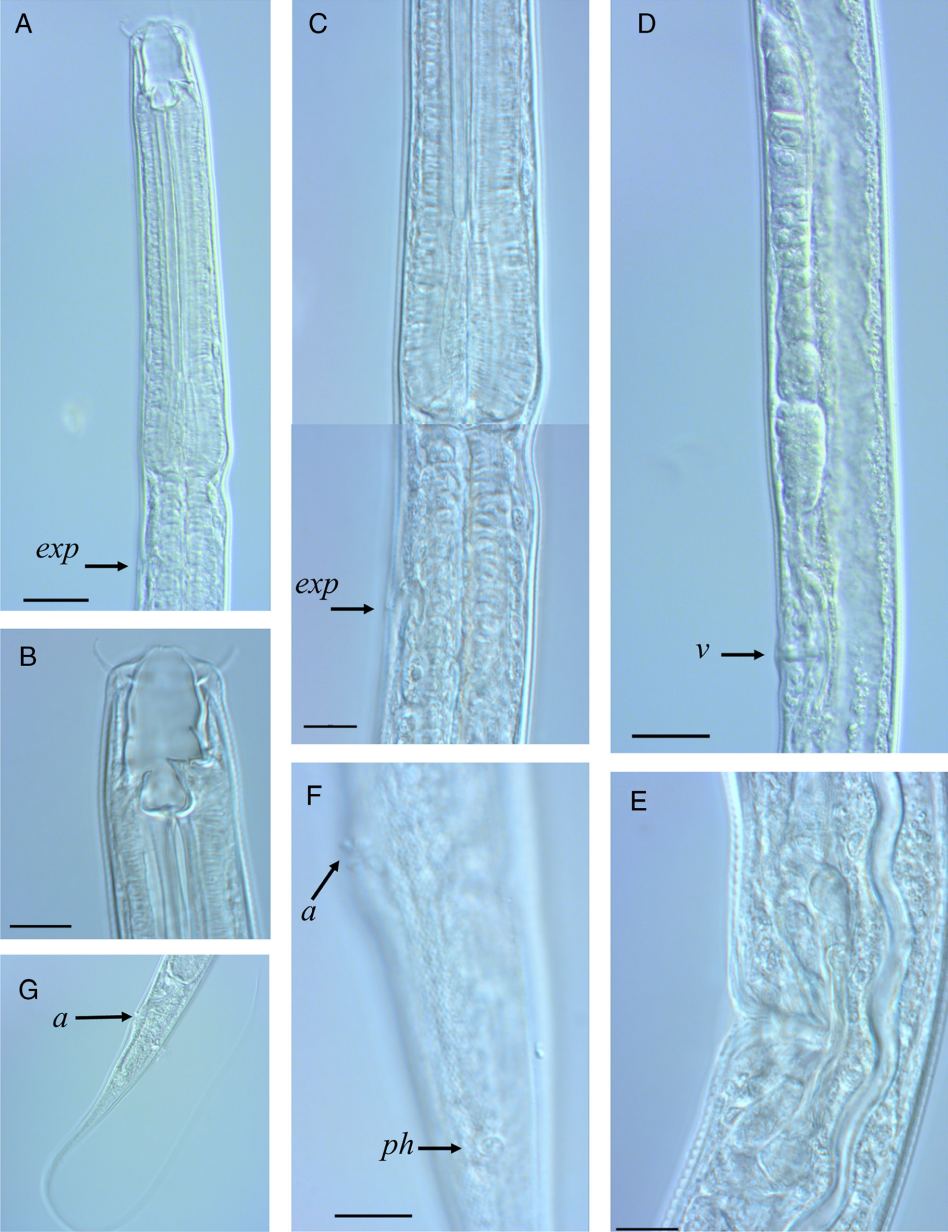
Light microscope pictures of females of *Butlerius butleri* Goodey, 1929 from South Africa. A: Anterior region with stoma, arrow indicating excretory pore; B: Stoma; C: Base of corpus showing swollen metacorpus and junction with isthmus, arrow indicating excretory pore; D: Vulval region and posterior genital tract; E: Vulval region showing schlerotised dorsal wall of uterus and four large glands; F: Tail region cuticle with punctations and position of phasmid; G: Tail. Scale bars: B, C, E, F: 10 µm; A, D, G: 20 µm.

**Figure 3:**
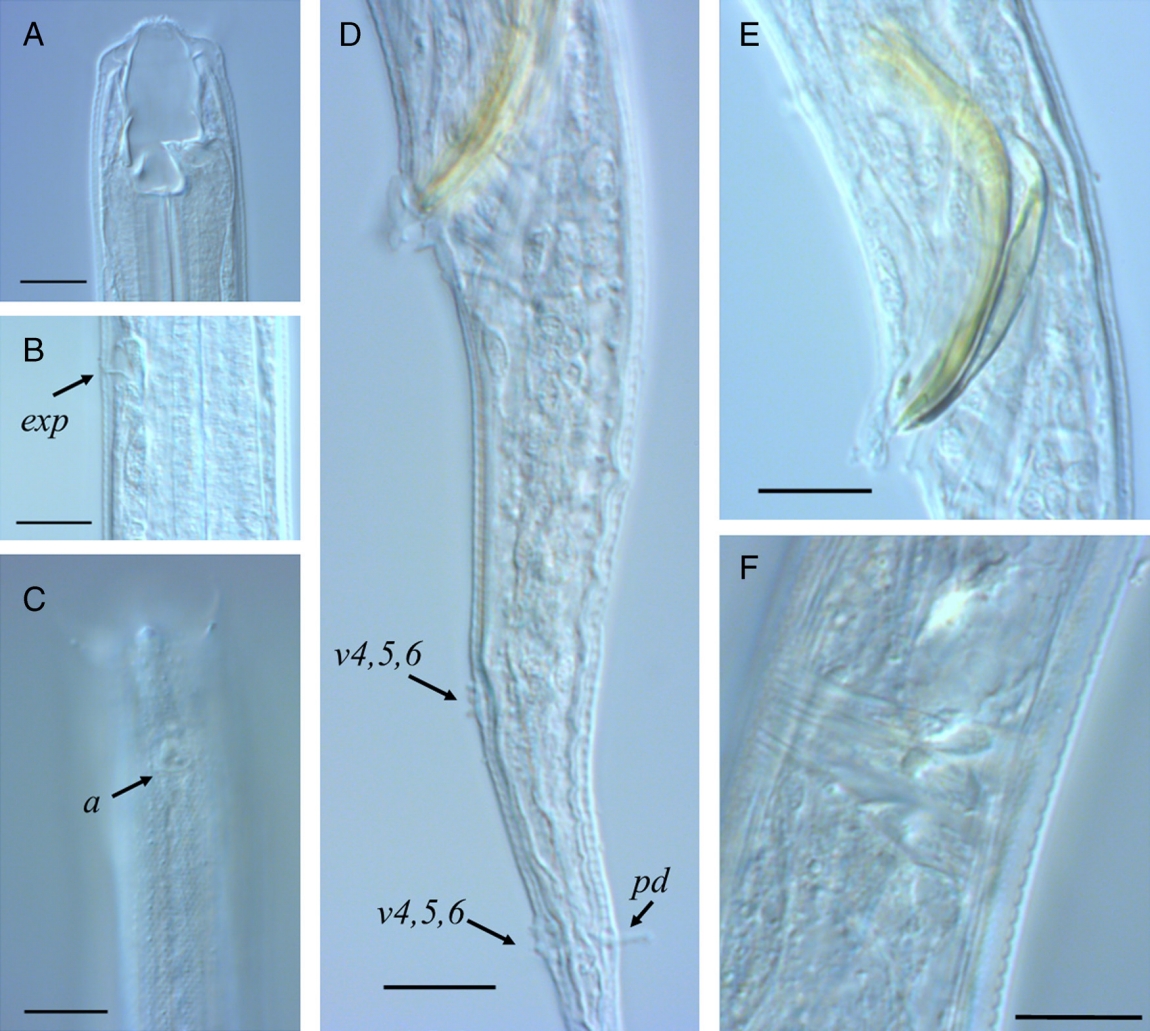
Light microscope pictures of males of *Butlerius butleri* Goodey, 1929 from South Africa. A: Stoma; B: Excretory pore; C: Anterior region showing cuticle with punctations and position of amphid; D: Cloacal region indicating cloacal flap, v5,6,7 clusters and posterior dorsal papilla; E: Cloacal region showing spicule and gubernaculum; F: Sphincter in mid-region of vas deferens. Scale bars: A to F: 10 µm.

Measurements: Table 2.

**Table 2.** Morphometrics of two populations of *Butlerius butleri* Goodey, 1929 females and males found in compost heaps in Potchefstroom, South Africa.

	Population 1		Population 2	
Characteristics	Female (*n* = 7)	Male (*n* = 10)	Female (*n* = 3)	Male (*n* = 2)
L	1,315 ± 90.2 (1,166-1,397)	1,193 ± 110.1 (1,082-1,365)	1,423.0*	–, 1,399
*L′*	979 ± 75.2 (880-1,086)	887 ± 68.2 (783-1,009)	1,140 ± 69.7 (1,093-1,220)	1,148, 1,136
*a*	44.5 ± 2.7 (40.8-47.6)	47.6 ± 6.0 (37.5-54.2)	39.9*	–, 43.4
*a′*	32.5 ± 2.2 (30.2-35.3)	35.3 ± 3.3 (29.3-39.6)	32.8 ± 1.9 (31.0-34.8)	36.8, 35.3
*b*	5.3 ± 0.4 (4.7-5.8)	5.2 ± 0.2 (4.9-5.4)	5.5*	–, 5.4
*b′*	3.9 ± 0.3 (3.5-4.1)	3.9 ± 0.2 (3.5-4.3)	4.3 ± 0.2 (4.1-4.5)	4.8, 4.4
*c*	4.6 ± 0.9 (4.0-6.0)	3.9 ± 0.4 (3.4-4.6)	4.5*	–, 5.3
*c′*	14.5 ± 2.0 (11.7-16.3)	13.5 ± 3.1 (8.7-16.9)	13.3*	–, 9.2
*V*/*T*	45.7 ± 1.3 (43.5-46.6)	39.2 ± 3.2 (36.3-44.4)	45.3*	–, 50.2
G1	12.6 ± 1.4 (11.4-14.8)	–	13.0*	–, –
G2	12.4 ± 0.9 (11.5-13.5)	–	13.8*	–, –
Body width at midbody	31 ± 3.1 (26-35)	25 ± 2.9 (20-30)	35 ± 1.1 (34-36)	31, 32
Labial region diameter	19 ± 1.9 (18-23)	17 ± 1.7 (14-20)	19 ± 0.7 (18-19)	18, 19
Cephalic setae length	8 ± 0.7 (8-9)	8 ± 0.9 (7-10)	7 ± 0.5 (7-8)	7, 7
Length of stoma	26 ± 1.5 (23-28)	24 ± 1.7 (21-27)	27 ± 0.6 (27-28)	26, 26
Stoma width	11 ± 1.1 (9-13)	10 ± 1.3 (8-12)	12 ± 0.9 (12-13)	12, 12
Cheilostom length	12 ± 0.8 (11-13)	10 ± 2.2 (5-13)	14 ± 1.6 (12-15)	13, 12
Gymnostom length	7 ± 1.0 (6-9)	7 ± 2.0 (5-10)	6 ± 0.4 (6-7)	6, 6
Stegostom length	7 ± 1.1 (6-9)	5 ± 1.1 (4-7)	7 ± 0.8 (6-8)	7, 8
Dorsal tooth length	9 ± 1.2 (7-10)	7 ± 0.9 (6-8)	7 ± 0.7 (6-7)	9, 7
Subventral tooth length	5 ± 0.9 (4-6)	4 ± 0.4 (4-5)	5 ± 0.7 (4-5)	5, 4
Corpus (procorpus and metacorpus)	137 ± 3.2 (133-141)	125 ± 7.2 (113-140)	144 ± 2.5 (142-147)	138, 134
Postcorpus (isthmus and basal bulb)	115 ± 5.3 (107-120)	102 ± 8.6 (86-118)	121 ± 5.2 (116-126)	102, 125
Pharynx (anterior end to base of basal bulb)	252 ± 8.1 (241-262)	227 ± 15.3 (199-258)	265 ± 6.4 (258-269)	240, 259
Excretory pore from anterior	191 ± 45.4 (163-243)	156 ± 9.4 (146-171)	157 ± 2.5 (155-158)	–, 173
Nerve ring from anterior	140 ± 6.3 (133-149)	123 ± 10.6 (100-140)	146 ± 3.5 (143-150)	138, 134
Metacorpus width	22 ± 2.5 (19-26)	20 ± 1.2 (18-22)	28 ± 3.7 (25-32)	20, 23
Basal bulb width	22 ± 2.6 (18-25)	18 ± 1.5 (16-21)	27 ± 0.6 (27-28)	18, 22
Cardia length	9 ± 1.3 (8-12)	9 ± 3.1 (6-14)	–	7, –
Anterior genital tract length	166 ± 27.8 (129-205)	–	196 ± 11.8 (185-209)	–
Posterior genital tract length	157 ± 15.2 (133-184)	–	188 ± 7.1 (182-196)	–
Body width at vulva	31 ± 3.2 (26-36)	–	35 ± 1.5 (34-36)	–
Vulva from anterior end	608 ± 42.5 (542-671)	–	683 ± 49.5 (645-739)	–
Vulva-anus distance	376 ± 32.1 (338-415)	–	457 ± 27.2 (427-481)	–
Vagina length	12 ± 2.5 (10-16)	–	14 ± 2.1 (12-16)	–
Rectum length	32 ± 2.6 (29-36)	–	31 ± 5.3 (28-37)	–
Body width at anus/cloaca	20 ± 1.6 (18-22)	24 ± 3.2 (19-28)	24 ± 0.4 (24-25)	26, 29
Testis length	–	496 ± 72.0 (392-589)	–	607, 703
Spicules length	–	40 ± 2.7 (36-43)	–	39, 43
Gubernaculum length	–	27 ± 2.6 (23-31)	–	30, 29
Tail length	290 ± 46.4 (225-325)	310 ± 43.2 (242-356)	317.0	–, 263
Phasmid posterior to anus	29 ± 1.5 (28-31)	36 ± 4.3 (30-44)	–	–, 37

**Notes:** All measurements in the form: mean±standard deviation (range). *L*′ = length from head to anus; *a′* = *L′* divided by width at midbody; – indicates structures that were not visible or not present; *indicates structure was only measured in one specimen of the population.

### Description

Female (*n* = 10): Body 1,166 to 1,423 µm long, slender, tapering posterior to anus. Cuticle with evenly spaced punctations. Lateral field marked by three lines. Labial region continuous with body contour, lips low, fused each with a setae 8 to 9 µm long. Stoma 23 to 28 µm long, 9 to 13 µm wide. Cheilostom barrel shaped, 11 to 15 µm long, cheilorhabdions anteriorly arched inwards. Gymnostom 6 to 9 µm long, connected to cheilostom by hyaline ligament. Stegostom anisomorphc, dorsal metastegostom bearing a prominent 7 to 10 µm long dorsal tooth, subventrally a smaller 4 to 6 µm sickle-shaped tooth. Dorsal pharyngeal gland opening on dorsal tooth. Pharynx diplogasteroid, 241 to 269 µm long. Corpus (procorpus + metacorpus) muscular, cylindrical, 133 to 147 µm long. Lumen of corpus heavily sclerotized. Metacorpus swollen, distinguished from procorpus by slightly more sclerotized lumen. Nerve ring situated at isthmus. Excretory pore situated posterior to nerve ring. Postcorpus (isthmus + basal bulb) 107 to 126 µm long, gradually expanding posteriorly. Cardia prominent, 8 to 12 µm long. Reproductive system didelphic, amphidelphic, both branches equal in length. Ovaries reflexed, oocytes arranged in single row except for multiple rows in growth zones. Spermatheca not well demarcated, uterus muscular, inner wall sclerotized dorsally. Four large glands opening into proximal part of uterus. Vagina narrow, 10 to 16 µm long, one-third of the corresponding body width. Vulva situated at 44 to 47% of body length, pore-like opening, not protruding. Rectum 28 to 37 µm long. Tail 225 to 325 µm long, filiform (broken in many specimens). Phasmid prominent, 28 to 31 µm posterior to anus.

Male (*n* = 12): similar to female, slightly smaller in size (1,082-1,399 µm). Reproductive system monorchid, testis 392 to 703 µm long, reflexed. Prominent sphincter present in mid-region of *vas deferens*. Spicules 36 to 43 µm long, slender, and curved, capitulum anteriorly flattened, circular margins, lamina narrow, ventrally curved. Gubernaculum 23 to 31 µm long, proximal part narrow, curved, handle-like, distal part rounded with a sleeve, distal tip bearing small, laterally directed, hook-like processes. Nine pairs of genital papillae, three pre-cloacal and six post-cloacal, formula: v1, v2, v3d/v4, ad, ph, v5, 6, 7, pd. The v5, 6, 7 clusters greatly separated, left subventral group at level of phasmid, right subventral group at level of posterior dorsal papilla. Anterior cloacal lip large, lobe-like, protruding posteriorly, bearing single prominent papilla, posterior lip bearing single pair of smaller papillae. Tail similar to female, 242 to 356 µm long, filiform.

#### Locality and material examined

Soil samples were collected from two home garden compost heaps (1: 26°42′25.3′′S 27°06′25.9′′E, 2: 26°41′17.4′S 27°06′05.6′E) in PotchefstroomNorth-West Province, South Africa. Slides containing 10 females and 12 males were deposited in the NCN (ARC-PHP, Biosystematics, Pretoria).

#### Diagnosis

The South African specimens of *B. butleri* are characterized by a 1,082 to 1,423 µm long body, cuticle with evenly spaced punctations, reproductive system didelphic, amphidelphic (*V* = 44-47%), both branches equal in length, four large glands opening into proximal part of uterus, males with prominent sphincter present in mid-region of vas deferens, spicules 36 to 43 μm long, gubernaculum 23 to 31 µm long , nine pairs of genital papillae, three pre-cloacal and six post-cloacal, formula: v1, v2, v3d/v4, ad, ph, v5, 6, 7, pd. The v5, 6, 7 clusters greatly separated, left subventral group at level of phasmid, right subventral group at level of posterior dorsal papilla. The two South African specimens were compared to the original description of *B. butleri* by Goodey (1929) as well as the redescription of the species by Ahmad et al. (2009) and a population reported by Shokoohi et al. (2015).

#### Relationships

The South African specimens of *B. butleri* compares well to the type population, however the following differences were observed: higher a values in both females and males (39.9-47.6 and 37.5-54.2 vs 18.3-23.3 and 19.1-19.6, respectively); higher V (44-47% vs 41%), and longer males (1,082-1,399 µm vs 970-1,150 μm) (Goodey, 1929). Ahmad et al. (2009) stated that the type specimens that were examined were flattened and may account for the low a value in the original description of the species. Ahmad et al. (2009) redescribed the species from specimensfound in decaying plant material from South Korea. Although smaller in size (based on most morphometric measurements) the South African specimens of the species is conspecific to the South Korean population. Apart from the smaller size, the following differences were observed: shorter body length in females (1,166-1,423 µm vs 1,335-1,857* *µm); and shorter tails in both females and males (225-325 µm and 242-356 µm vs 361-570 µm and 336-503 μm, respectively) (Ahmad et al., 2009). The South African specimens were also compared to the most recent description of the species from Iran (Shokoohi et al., 2015). The following differences were observed in females: higher a value (39.9-47.6 vs 34.0-41.2); lower V (44-47% vs 45-51%); larger dorsal tooth (7-10 µm vs 4-5 µm); shorter vagina (10-16 µm vs 15-21) and a shorter tail (225-325 µm vs 304-410 µm in the Iranian population). In males the following differences were observed: shorter body length (1,082-1,399 µm vs 1,266-1,533 µm in Iranian population); smaller dorsal tooth (6-8 µm vs 4.8 µm); shorter spicules (36-43 µm vs 44-47 µm) and shorter gubernaculum (23-31 µm vs 33-37 µm).

In comparison to the other species of the genus the South African populations of *B. butleri* can be separated from the following species due to the absence of longitudinal striae (absent in *B. butleri* vs present in the listed species): *B. canadensis* Ebsary, 1986, *B. degrissei* Grootaert and Jaques, 1979, *B. gerlachi* Meyl, 1957, *B. macrogubernaculum* (Chitambar, 1990) Sudhaus and Fürst von Lieven, 2003, and *B. micans* Pillai and Taylor, 1968. As a didelphic species, *B. butleri* can be distinguished from the monodelphic species: *B. degrissei*, *B. kaplini* (Ryss, 1989) Sudhaus and Fürst von Lieven, 2003,*B. macrospiculum* Hunt, 1980, *B. macrogubernaculum*, and *B. monhystera* Taylor, 1964. *Butlerius butleri *differs from *B. longipype* (Khera, 1969) Sudhaus and Fürst von Lieven, 2003 in being the larger of the two species withdifference is the following for both females and males: *L* (1,166-1,423 µm vs 1,000-1,200 µm in females; 1,082-1,399 µm vs 750-850 µm in males); *a* value (39.9-47.6 vs 25.0-28.0 in females; 37.5-54.2 vs 26.0-28.0 in males); *b* value (4.7-5.8 vs 6.5-6.8 in females; 4.9-5.4 vs 6.5-6.8 in males); *c* value (4.0-6.0 vs 3.1-3.5 in females; 3.4-5.3 vs 3.0-3.3 in males); position of the vulva (*V* = 44-47% vs 40-43%); length of the spicules (36-43 µm vs 30-32 µm) and length of gubernaculum (23-31 µm vs 12-13 µm). The South African populations of *B. butleri* can be distinguished from *B. spirifer* (Skwarra, 1921) Zullini and Loof, 1980 based on the number ofpre-clocacal papillae (three in *B. butleri* vs one in *B. spirifer*) and body length of both males and females (1,166-1,423 µm vs 1,000 µm in females; and 1,082-1,399 µm vs 890 µm in males). *Butlerius butleri* most closely resembles *B. demani* (Schneider, 1923) Andrássy, 1984 and *B. okai* Rahm, 1938, however can be separated from *B. demani* by the presence of males (present in *B. butleri* vs absent in *B. demani*) and from *B. okai* by the presence of a gubernaculum (present in *B. butleri* vs absent in *B. okai*).

### Phylogenetic analyses

The Nucleotide BLAST (Blastn) search based on the partial SSU sequences (MN710517 and MN710518) showed > 99% similarity to a *B. butleri* from Iran (accession no. KP453998). Blastn search using the partial LSU sequences of our populations (MN710521 and MN71522) revealed 92% similarity to a *Diplogastrellus* sp. from Germany (accession no. KJ877248).

**Figure 4:**
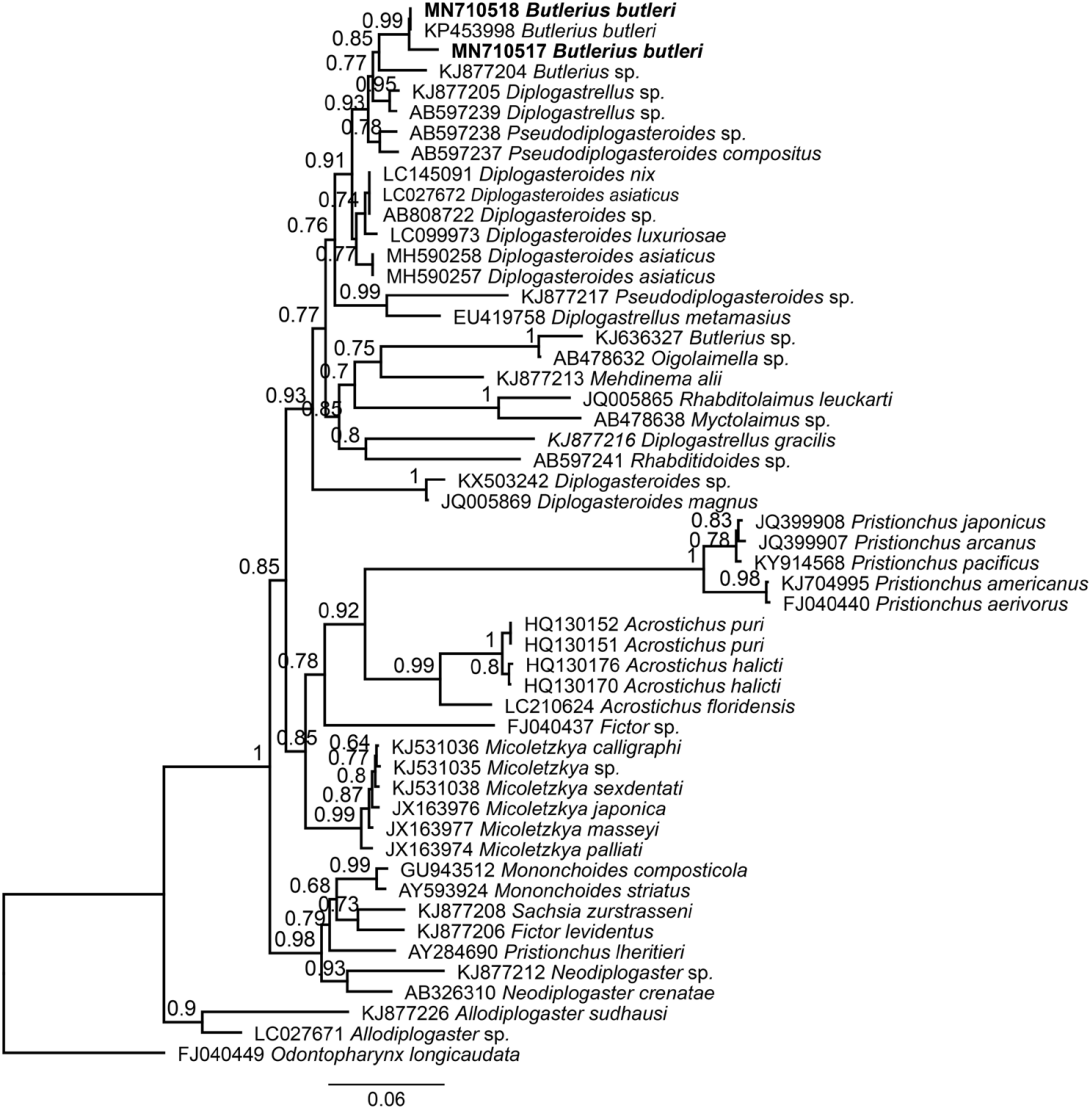
Bayesian phylogenetic tree with 50% majority rule of *Butlerius butleri* from South Africa using small subunit (SSU) rDNA gene sequences under GTR + G model (lnL = 7,712.8957; K = 117; freqA = 0.2568; freqC = 0.1991; freqG = 0.2542; freqT = 0.2900; rAC = 0.9137; rAG = 2.6380; rAT = 1.9015; rCG = 0.2936; rCT = 4.5476; rGT = 1.0000; gamma shape = 0.3330). The newly obtained sequences are indicated by bold font.

**Figure 5:**
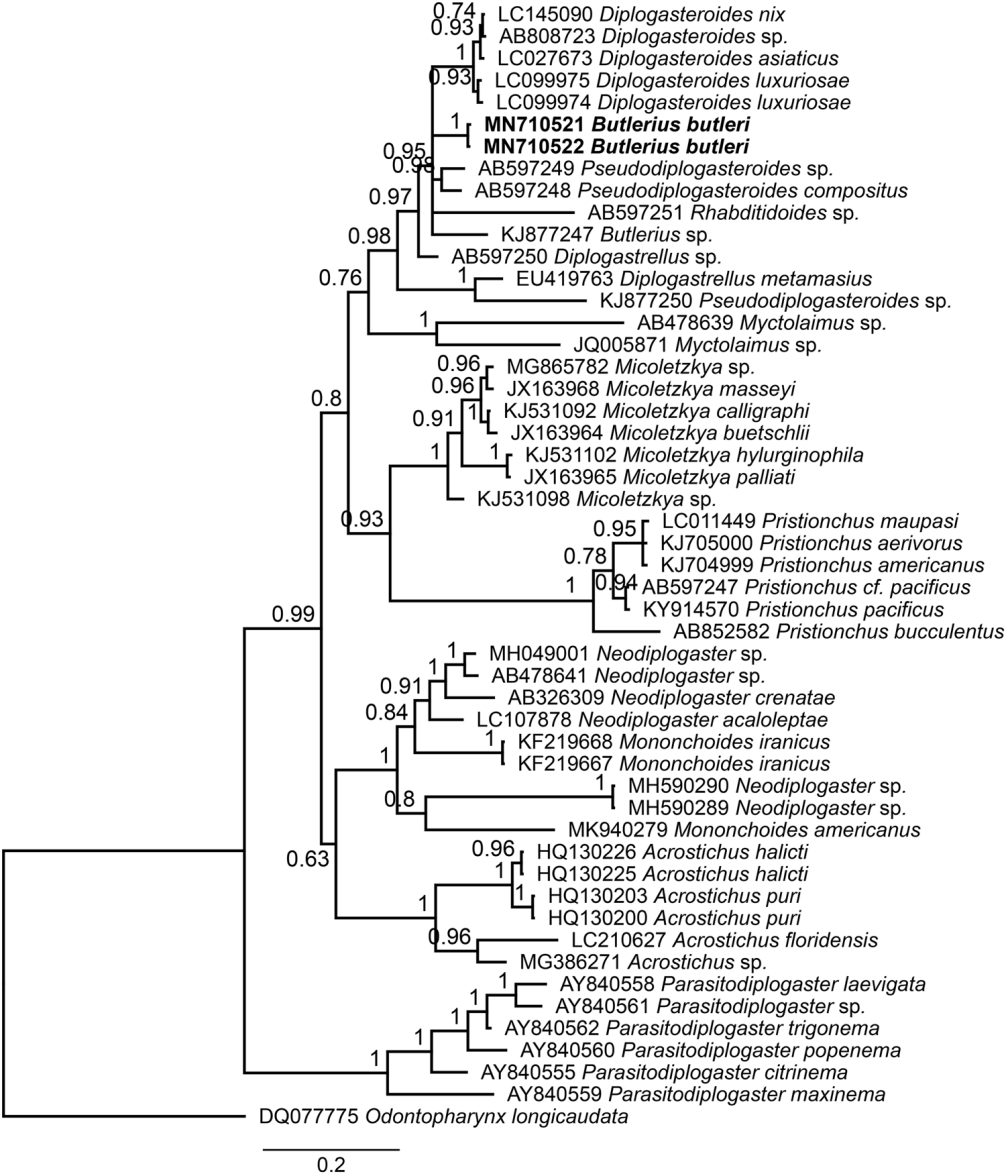
Bayesian phylogenetic tree with 50% majority rule of *Butlerius butleri* from South Africa using Large subunit (LSU) rDNA gene sequences under GTR + I + G model (lnL = 11,826.2635; K = 111; freqA = 0.1404; freqC = 0.2230; freqG = 0.3544; freqT = 0.2822; rAC = 0.8351; rAG = 2.7011; rAT = 1.4534; rCG = 0.5981; rCT = 4.6690; rGT = 1.0000; gamma shape = 0.5020). The newly obtained sequences are indicated by bold font.

Bayesian topology based on partial SSU sequences confirmed the well-supported sister relationship (PB: 0.99) of South African populations (MN710517 and MN710518) with another population of *B. butleri* from Iran (KP453998) (Fig. 4). Moreover, these populations formed a highly supported group (BP: 0.93) with two unidentified populations of *Diplogastrellus* Paramonov, 1952 (KJ77205 and AB597239), two *Pseudodiplogasteroides* Körner, 1954 populations (AB597237 and AB597238) and an unidentified population of *Butlerius* (KJ877204). Bayesian inference tree using the D2-D3 LSU sequences showed that two newly sequenced populations of *B. butleri* are closely related and clustered (BP: 0.84) with two populations of *Pseudodiplogasteroides* (AB597248 and AB597249) and an unidentified population of *Rhabditidoides *Rahm, 1928 (AB597251) (Fig. 5).

## Discussion

Two populations of *Butlerius* were collected in 2019 from garden compost in Potchefstroom, South Africa and characterized as *B. butleri*. Although differences were observed between the South African populations and the other populations of *B. butleri* reported previously, the South African specimens were conspecific to the species and represent the first report of the species *B. butleri* in South Africa. The populations of *B. butleri* described herein broaden the morphometric range of the species as the South African populations measured smaller than both the South Korean and Iranian populations of the species. The constructed phylogenetic tree based on SSU sequences confirmed the phylogeny published by Shokoohi et al. (2015) where *Butlerius* populations represent a monophyletic group and were closely related to the genera *Diplogatrellus* Paramonov, 1952 and *Pseudodiplogasteroides* Körner, 1954.However, *Butlerius* can be distinguished from *Diplogastrellus* based on the shape of the stoma (barrel shaped in *Butlerius* vs tube shaped in *Diplogastrellus*) and length of the cephalic setae (longer in *Butlerius* as compared to *Diplogastrellus*). Furthermore, *Butlerius* could be separated from *Pseudodiplogasteroides* based on the shape of the stoma (barrel shaped in *Butlerius* vs tube shaped in *Pseudodiplogasteroides*) and the presence of remnants of haustrulum in the terminal pharyngeal bulb of *Pseudodiplogasteroides* that is not present in *Butlerius*. The evolutionary relationship of *B. butleri* based on partial LSU sequences revealed a close relation of this genus and *Diplogasteroides* De Man, 1912, however, these genera are differentiated by the shape of the stoma (barrel shaped in *Butlerius* vs tube shaped in *Diplogasteroides*) and the shape of the dorsal tooth in the stegostom (thorn-like in *Butlerius* vs dorsal tooth formed by three rods, lateral ones distally diverging in *Diplogasteroides*). Despite of novel phylogenetic relationships of *B. butleri* based on partial LSU rDNA sequence, monophyly of the genus could not be solely confirmed due to lack of sequences for the other members of the genus in the GenBank. Ultimately, adding further molecular data to the current phylogeny in the future would improve our knowledge and provide better resolution on taxonomy of the genus.
